# Adult-onset Still’s Disease in a Female Patient with Schizophrenia: A Case Report and Literature Review

**DOI:** 10.7759/cureus.3019

**Published:** 2018-07-21

**Authors:** Nasim Golchin, Mohaddeseh Sharifzadeh, Mina Fransawy Alkomos, Issac Sachmechi

**Affiliations:** 1 Endocrinology, Icahn School of Medicine at Mount Sinai/Queens Hospital Center, New York, USA; 2 Internal Medicine, Icahn School of Medicine at Mount Sinai Queen Hospital Center, New York, USA; 3 Research, California Institute of Behavioral Neurosciences & Psychology, Sacramento, USA; 4 Internal Medicine, Icahn School of Medicine at Mount Sinai/Queens Hospital Center, New York, USA

**Keywords:** still's disease, aosd, interleukin 6, schizophrenia, juvenile rheumatoid arthritis, autoimmune disease, inflammation

## Abstract

Adult-onset Still’s disease (AOSD) is a rare diagnosis. In small percentage of cases, AOSD is associated with other autoimmune diseases including schizophrenia. Despite the lack of sufficient studies, both conditions may share similar autoimmune pathogenic pathways.

Herein we describe a 36-year-old woman with the past medical history of schizophrenia who presented with spiking fevers, arthralgia, evanescent rash and pleural chest pain. She reported developing these symptoms a while after poor compliance with her antipsychotic medication. On admission, physical examination was remarkable for high-grade fever, maculopapular rash, oligo arthralgia, hepatomegaly and lymphadenopathy. Laboratory investigation revealed leukocytosis with neutrophilia and markedly elevated ferritin. The patient met four out of four major, and three out of five minor Yamaguchi criteria for AOSD. The patient started on therapy with corticosteroid. Soon after, her symptoms resolved and most of her biochemical markers went back to normal.

We review the literature on co-existence of AOSD with other autoimmune diseases, we also discuss that there may be a correlation between ceasing antipsychotic medication (with known immunomodulatory effect) in a schizophrenic patient and triggering an auto-inflammatory process such as AOSD in a susceptible host. In addition, we discussed the possible similar autoimmune pathway of schizophrenia to pathogenesis of AOSD.

## Introduction

Adult-onset Still's disease (AOSD) is a rare systemic auto-inflammatory disorder of unknown etiology and pathogenesis [1-2]. The clinical presentation has a wide spectrum of manifestations from a characteristic triad of a sudden onset of spiking fevers with an evanescent rash and arthritis or arthralgia to critical complications such as disseminated intravascular coagulopathy (DIC), diffuse alveolar hemorrhage, macrophage activating syndrome (MAS), hepatic failure, hemophagocytic lymphohistiocytosis (HLH) [3]. To date, no serological markers have been specified for AOSD. Among different diagnostic criteria which have been published, Yamaguchi Criteria [2] has been commonly used being the most sensitive. Depending on different parameters including underlying condition, severity of disease or level of responsiveness to empirical treatment, AOSD management varies from anti-inflammatory agents (nonsteroidal anti-inflammatory drugs (NSAIDS), corticosteroids), immune-suppressants and rheumatologic agents (methotrexate, azathioprine, tacrolimus, and cyclosporine) to intravenous immunoglobulin (IVIG), anti-TNF-α and anti-interleukins for treatment of refractory AOSD.

Schizophrenia is a heterogeneous neuropsychiatric disorder associated with persistent psychosocial disability that can affect up to one percent of the population worldwide. Increased levels of pro-inflammatory markers and cytokines in cerebrospinal fluid and peripheral blood in the patient with schizophrenia [4] supports the role of immune-inflammatory factors in pathophysiology of the disease with similar course in autoimmune diseases which is supported in plenty of literatures. Lack of insight and chronicity in the course of schizophrenia results in poor compliance with the treatment. Here, we report an interesting rare case of AOSD in a female patient with past medical history of schizophrenia treated with antipsychotic medication who met all major and four out of five minor Yamaguchi criteria [2].

## Case presentation

A 36-year-old female with past medical history significant for schizophrenia presented to the hospital after experiencing arthralgia for nine days followed by an evanescent rash for three days accompanied by persistent high-grade fever. Her symptoms were associated with pleuritic chest pain. The rash was non-pruritic and non-painful spreading over the neck, trunk, and all four extremities. The patient was diagnosed with schizophrenia five years before to her admission, and has been receiving olanzapine 20 mg daily for the last six months. She admitted noncompliance with her medication recently, due to developing diabetes mellitus and weight gain while being on olanzapine.

In the emergency department, her initial vital signs were as follows: temperature, 103.7°F (39.8°C); blood pressure, 111/55 mmHg; heart rate, 141 beats/minute; and respiratory rate 22 breaths/minute. The patient looked anxious and diaphoretic. Skin examination revealed salmon-like, blanchable, maculopapular rash of various shapes and sizes, most prominent over bilateral extremities. Soft, tender and mobile lymph nodes were palpated in the left cervical and left submandibular chains. Joint examination revealed reduced range of motion of both shoulders, right elbow, left wrist and right third proximal interphalangeal (PIP) joints. Her cardiac and pulmonary examination discovered no abnormalities.

Table 1 describes the laboratory examination results at the presentation.

**Table 1:** Laboratory values at the presentation.

Laboratory parameter	Patient value	Reference range
Leukocyte count (x10^9^/L)	23.2	4.5-11
Neutrophil (%)	81.0	40-74
Hemoglobin (g/dL)	11.9	12-19
Hematocrit (%)	34.6	37-47
Platelet (x10^9^/L)	340	130-400
Alkaline phosphate (U/L)	49	45-115
Aspartate aminotransferase (U/L)	18	8-40
Alanine aminotransferase (U/L)	11	8-40
Gamma-glutamyltransferase (U/L)	22	9-40
Lactate dehydrogenase (U/L)	165	100-250
Albumin (g/dL)	2.9	3.5-5.5
Protein (g/dL)	5.7	6.0-7.8
Total bilirubin (mg/dL)	0.8	0.1-1.0
Glucose, serum	150	<120
Hemoglobin A1c	7.1	<5.7

On admission radiograph of the chest revealed normal cardiac silhouette without any pleural effusions or pulmonary infiltration. Vancomycin and ceftriaxone were empirically started which were discontinued soon after the admission because the symptoms were not consistent with a bacterial infection, the patient then was managed symptomatically with acetaminophen and intravenous fluids. Over the next 36 hours, the patient continued to have spiking fevers with negative blood/urine cultures. Abdominal ultrasound revealed hepatomegaly and echocardiogram revealed trace pericardial effusion. On hospital day three, empiric gatifloxacin was started. Spiking fever persisted on following days four, five and six. Numerous lab studies including blood cultures and urine culture were performed to rule out infectious possibilities, e.g., antibody assays for rubella, mumps, cytomegalovirus, Epstein‐Barr virus, parainfluenza, Coxsackievirus, adenovirus, influenza, human herpesvirus 6, parvovirus B19, hepatitis B and hepatitis C, Mycoplasma pneumoniae, Chlamydia pneumoniae, Borrelia burgdoferi, Quantiferon test, Pneumococcal and Legionella urinary antigens, Chlamydia, Mycoplasma and HIV. All of which proved to be negative. Lymph node biopsy has been done, reactive benign lymphadenopathy was reported. Infectious diseases consultation advised initiation of vancomycin despite lack of infectious source.

Table 2 describes laboratory examination as per rheumatology consultation.

**Table 2:** Rheumatologic parameters. ANA: Antinuclear antibody; Anti-ds-DNA: Anti-double stranded DNA; Anti-La/Anti-SSB: Anti-Sjogren’s-syndrome related antigen B; Anti-Ro/Anti-SSA: Anti-Sjogren’s-syndrome related antigen; Anti-Sm Ab: Anti-smith antibodies; Anti-RNP: Anti-ribonucleoprotein; C-ANCA: Cytoplasmic antineutrophil cytoplasmic antibodies; P-ANCA: Perinuclear anti-neutrophil cytoplasmic antibodies; RF: Rheumatoid factor; ESR: Erythrocyte sedimentation rate; CRP: C-reactive protein; VDRL: Venereal disease research laboratory.

Laboratory parameter	Patient value	Reference range
Ferritin (ng/dL)	2451.7	10-230
VDRL	Non-reactive	
ESR (mm/hr)	124	0-20
CRP (mg/L)	150	0-10
ANA	Negative	
Anti-ds DNA	Negative	
Anti-Ro, Anti-La	Negative	
Anti-Sm Ab	Negative	
P-ANCA, C-ANCA	Negative	
Anti-RNP	Negative	
RF (IU/ml)	<20	0-20

Based on Yamaguchi criteria, the patient was diagnosed to have AOSD, hence steroids with 50 mg Solu-Medrol intravenously started. Over the next four days, fever, rash and arthralgia resolved and serum ferritin levels decreased to 1085.2 ng/mL. The patient was discharged apyretic, in good clinical condition on oral prednisone and advised to follow up in outpatient clinic.

## Discussion

We reported a case of AOSD in a patient with known history of schizophrenia after discontinuation of her antipsychotic medication. According to Yamaguchi criteria [2] she met four out of four major criteria, including fever >39°C, lasting longer than one week, arthralgia and arthritis, lasting longer than two weeks, evanescent rash, and leukocytosis of 23,000/mm^3^ with >80% polymorphonuclear cells, as well as three out of five minor criteria including recent development of left cervical and submandibular lymphadenopathy, hepatomegaly, and negative tests for antinuclear antibody and rheumatoid factor. Exclusion criteria were thoroughly evaluated and results were negative for viral or bacterial infection, malignant lymphoma, and other rheumatic diseases. Furthermore, serum ferritin level was increased more than five-fold suggestive of AOSD [5], which in some studies has been considered to be associated with dysregulation of cytokines [6].

To the best of our knowledge, this was the second case of schizophrenia who was presenting with AOSD. The first reported case [7] was a 31-year-old man with past medical history significant for Crohn’s disease for 12 years and schizophrenia for 10 years presented with spiking fever, arthritis, skin rash, hepato-splenomegaly and pleural effusion diagnosed with AOSD.

Despite all the scientific evidence, the etiology of AOSD remains unknown [1-2]. Several hypotheses have been proposed for pathophysiology of Still's disease, among them, the reactive syndrome has been very popular, where viral and microbial infectious agents can initiate the disease in some genetically susceptible patients. Several viral and microbial entities have been reported in small case reports and series including rubella, mumps, echovirus, cytomegalovirus, Epstein‐Barr virus, parainfluenza, Coxsackievirus B4, adenovirus, influenza A, human herpesvirus, parvovirus B19, hepatitis B and hepatitis C, Mycoplasma pneumoniae, Chlamydia pneumoniae, Yersinia enterocolitica, Brucella abortus, and Borrelia burgdoferi [2, 8-10]. In more recent studies predominance of T helper 1 cytokines, e.g., tumor necrosis factor alpha (TNF-α), interferon gamma (INF-γ) and also other cytokines including interleukin 6 (IL-6), and interleukin 18 (IL-18) in the peripheral blood and pathological tissues of untreated patient with active AOSD has been reported which shows the importance of innate and adaptive immunity in pathogenesis of AOSD [11].

On the other hand in patients with schizophrenia pro-inflammatory cytokines has been shown to be overproduced in cerebrospinal fluid and peripheral blood [4], although there is no definite immune marker to evaluate the neuroprogression of schizophrenia, increased markers of cellular immunity such as IL-6 [12] and significant decrease in IL-6 after antipsychotic treatment in patients with first episode and relapsed schizophrenia [13] which by itself can predispose the patient to obesity and metabolic disorders can support the immune mediation in pathogenesis of schizophrenia.

Our assumption is that while taking the patient off the immunomodulatory effect of olanzapine may predispose the patient to develop AOSD, therefore a possible pathophysiologic link between the AOSD and schizophrenia may be suggested. Also, it has been known that autoimmune diseases have the tendency to appear in clusters. There are reports of AOSD accompanying other autoimmune disorders including this case.

We have reviewed articles which AOSD has been associated with other autoimmune diseases (see Table 3).

**Table 3:** Characteristics of patients with AOSD and associated autoimmune disease (a review of literature). AST: Aspartate aminotransferase; AIH: Autoimmune hepatitis; ALP: Alkaline phosphatase; ALT: Alanine aminotransferase; ANA: Antinuclear antibody; Anti-CCP: Anti-cyclic citrullinated peptide; Anti-ds-DNA: Anti-double stranded DNA; Anti-La/Anti-SSB: Anti-Sjogren’s-syndrome related antigen B; Anti-LKM-1: Anti-liver-kidney microsomal antibody; Anti-Ro/Anti-SSA: Anti-Sjogren’s-syndrome related antigen; AOSD: Adult-onset Still’s disease; AST: Aspartate aminotransferase; C3: Complement 3; C4: Complement 4; CD: Crohn disease; CH50: Total complement activity; CRP: C-reactive protein; ESR: Erythrocyte sedimentation rate; FT3: Free triiodothyronine serum; FT4: Free thyroxin serum; Hb: Hemoglobin; IBD: Irritable bowel disease; LAD: Lymphadenopathy; LDH: Lactate dehydrogenase; LFT: Liver function test; NA: Not available; Ne: Neutrophil granulocytes; Neg: Negative; RF: Rheumatoid factor; SRA: Seronegative rheumatoid arthritis; TGAb: Thyroglobulin autoantibodies; TSH: Thyroid-stimulating hormone; WBC: White blood cells; WNL: Within normal limits.

Study	Sex	Associated disease	Lab test	Symptoms on admission	Age	Order of occurrence
Hu et al. [13]	F	Graves’ disease	FT3 & FT4: WNL TGAb (+) Low TSH C3, C4: WNL ANA & RF (-) Neu (86%)	Irritability, Fatigue, Weight loss, LAD, Splenomegaly, Spiking fever, Arthralgia, Evanescent rash	43	Co-existence
Niranvichaiya & Triwongwaranat [14]	M	SRA	WBC: 29,020 Neu: 82% Ferritin: >100,000 Elevated LFT & ESR Neg RF, Anti CCP, ANA & ds-DNA LDH: 6,883	High fever, Rash, Arthralgia, Pharyngitis	36	SRA prior to AOSD
Kono et al. [7]	F	Schizophrenia & CD	WBC: 18,900/ml, Neu: 88% CRP: 29.4 mg/dL ESR: 119.2 mm/1h Blood culture: Neg	Arthritis, fever, skin rash followed by hepatosplenomegaly and pleural effusion	31	Schizophrenia and CD prior to AOSD
Rajabally et al. [15]	F	CD	Neu: 19,0000 ESR: 100 CRP: 190 Ferritin: 20,380 Neg RF, ANA & Anti CCP	Fever, arthralgia, pharyngitis, rash without gastrointestinal symptoms to suggest a flare up of IBD	30	CD prior to AOSD
Katsanos et al. [16]	M	CD	Hb: 12.3 gr/dl WBC: 16,200/mm^3^ ESR: 27 mm/h CRP 14 mg/dl	Bloody diarrhea	38	AOSD prior to CD
Bozek et al. [17]	M	Autoimmune meningoencephalitis	NA	Headaches, Visual disturbances, Fever, Fatigue and cognitive decline	31	Co-existence
Fujii et al. [18]	F	AIH	*Prior to AOSD:* AST: 1,040 IU/L ALT: 1,124 IU/L LDH: 615 IU/L ALP: 821 IU/L Ferritin: 3,043 ng/ml After AOSD: WBC: 13,300 Neu > 80% AST: 69 IU/L ALT: 81 IU/l LDH: 462 IU/l ALP: 216 IU/L ESR: 47 mm/h CRP: 4.41 mg/dl Ferritin: 18,306 ng/ml CH50: 63.8 u/ml Neg Anti-Ro, Anti-La, Anti-Sm & Anti LKM-1	High fever (39°C>2Ws), Oligo arthralgia, Salmon pink maculopapular erythema, Koebner phenomenon, Cervical LAD	17	AIH prior to AOSD

## Conclusions

Coexistence of AOSD and schizophrenia has been rarely reported and it is difficult to make a conclusive clear correlation, however, there may be some interactions through their common autoimmune pathogenesis. Although our study cannot rule out the coincidental link, we are describing the possibility of poorly controlled autoimmune process which can lead to the flare of another autoimmune in the susceptible host. More studies on possible pathophysiological links may reveal the association of AOSD with other autoimmune diseases including schizophrenia. Furthermore, maintaining a high index of clinical suspicion for this condition in patients presenting with fever, evanescent rash, and arthralgia may expedite the diagnosis and appropriate treatment for AOSD and prevent the progression of the disease to life-threatening conditions.

## References

[1] Efthimiou P, Paik PK, Bielory L (2006). Diagnosis and management of adult onset Still’s disease. Ann Rheum Dis.

[2] Yamaguchi M, Ohta A, Tsunematsu T (1992). Preliminary criteria for classification of adult Still’s disease. J Rheumatol.

[3] Bürgi U, Mendez A, Hasler P, Hüllstrung HD (2012). Hemophagocytic syndrome in adult-onset Still’s disease (AOSD): a must for biologics? Case report and brief review of the literature. Rheumatol Int.

[4] Khandaker GM, Cousins L, Deakin J, Lennox BR, Yolken R, Jones PB (2015). Inflammation and immunity in schizophrenia: implications for pathophysiology and treatment. Lancet Psychiatry.

[5] Rosário C, Zandman-Goddard G, Meyron-Holtz EG, D'Cruz DP, Shoenfeld Y (2013). The hyperferritinemic syndrome: macrophage activation syndrome, Still’s disease, septic shock and catastrophic antiphospholipid syndrome. BMC Med.

[6] Mehta B, Efthimiou P (2012). Ferritin in adult-onset Still’s disease: just a useful innocent bystander. Int J Inflam.

[7] Kono M, Oshitani N, Sawa Y (2003). Crohn’s disease complicated by adult-onset Still’s disease. J Gastroenterol.

[8] Wouters JM, van der Veen J, van de Putte LB, de Rooij DJ (1988). Adult onset Still’s disease and viral infections. Ann Rheum Dis.

[9] Perez C, Artola V (2001). Adult Still’s disease associated with Mycoplasma pneumoniae infection. Clin Infect Dis.

[10] Mavragani CP, Spyridakis EG, Koutsilieris M (2012). Adult-onset Still’s disease: from pathophysiology to targeted therapies. Int J Inflam.

[11] Müller N (2017). Neuroprogression in schizophrenia and psychotic disorders: the possible role of inflammation. Mod Trends Pharmacopsychiatry.

[12] Borovcanin M, Jovanovic I, Radosavljevic G, Djukic Dejanovic S, Stefanovic V, Arsenijevic N, Lukic ML (2013). Antipsychotics can modulate the cytokine profile in schizophrenia: attenuation of the type-2 inflammatory response. Schizophr Res.

[13] Hu Y, Wang H, Deng J (2014). Adult-onset Still’s disease associated with thyroid dysfunction: case report and review of the literature. Open Rheumatol J.

[14] Niranvichaiya S, Triwongwaranat D (2017). Diagnostic challenge: a report of two adult-onset Still’s disease cases. Case Rep Dermatol Med.

[15] Rajabally MN, Watermeyer GA, Levin DA (2010). A case of Crohn’s disease complicated by adult onset Still’s disease. J Crohns Colitis.

[16] Katsanos KH, Siozopoulou V, Sigounas D, Tsianos VE, Christodoulou D, Mitsi V, Tsianos EV (2013). Adult-onset Still’s disease preceding Crohn’s disease. J Crohns Colitis.

[17] Bożek M, Konopko M, Wierzba-Bobrowicz T, Witkowski G, Makowicz G, Sienkiewicz-Jarosz H (2017). Autoimmune meningitis and encephalitis in adult-onset still disease - case report. Neurol Neurochir Pol.

[18] Fujii K, Rokutanda R, Osugi Y, Koyama Y, Ota T (2012). Adult-onset Still’s disease complicated by autoimmune hepatitis: successful treatment with infliximab. Intern Med.

